# Pathogenesis of pseudorabies virus in mouse placenta: insights into maternal–infant transmission mechanisms

**DOI:** 10.1128/spectrum.03648-25

**Published:** 2026-03-24

**Authors:** Han-Yu Li, Tong Xu, Xin Wu, Bao-Ling Liu, Yi-Xin Yan, Li-Na Shao, Bing-Zhou Huang, Yi Qing, Liang-Peng Ge, Zuo-Hua Liu, Jing Sun, Xiu Zeng, Zhi-Wen Xu, Ling Zhu

**Affiliations:** 1 College of Veterinary Medicine, Sichuan Agricultural University 506176 , Chengdu, China; 2 Chengdu Livestock and Poultry Genetic Resources Protection Center, Chengdu, Sichuan, China; 3 Chongqing Academy of Animal Sciences 580592, Chongqing, China; 4 National Center of Technology Innovation for Pigs, Chongqing, China; 5 Key Laboratory of Animal Diseases and Human Health of Sichuan Province, Chengdu, China; Thomas Jefferson University, Philadelphia, Pennsylvania, USA

**Keywords:** pseudorabies virus, placenta, inflammation, IUGR, vertical transmission

## Abstract

Pseudorabies virus (PRV) infection poses a significant threat to reproductive success in pregnant pigs, often leading to abortion and stillbirth. Systematic studies on the vertical transmission mechanism of PRV through the placenta remain limited. To address this research gap, we developed a PRV infection model in pregnant mice. Immunohistochemical analysis detected strong PRV-positive signals in placental trophoblasts and fetal brains, with viral loads significantly higher than those in the maternal bloodstream. These results suggest that PRV can infect the placenta via maternal circulation and subsequently be transmitted to the fetus. Further analysis revealed that PRV infection disrupts placental vascular architecture and compromises the integrity of tight junction proteins (TJPs) ZO-1 and occludin, facilitating viral entry into fetal tissues. Transcriptomic profiling of placental tissues showed activation of inflammatory responses, oxidative stress, and cell death pathways. Concurrently, genes involved in hormone biosynthesis, angiogenesis, and trophoblast proliferation and differentiation were markedly downregulated. Together, these results indicate that PRV compromises placental integrity in mice through a combination of physical barrier disruption, inflammatory microenvironment formation, and vascular dysfunction. This provides a reference for the mechanism of vertical transmission of PRV through the placenta in mice.

**IMPORTANCE:**

Pseudorabies virus can be transmitted vertically by disrupting the barrier function of the placenta. Maternal viremia drives preferential PRV replication within the placenta. PRV preferentially colonizes decidual and labyrinthine placental regions. PRV can cause pregnancy failure via inflammation.

## INTRODUCTION

Pseudorabies (PR), also known as Aujeszky’s disease, was first described in the United States in 1813 and has since become a globally distributed infectious disease, resulting in substantial economic losses to the swine industry over the past decades (1). The etiological agent, porcine pseudorabies virus (PRV), is officially classified as Suid herpesvirus 1 (SuHV-1) and belongs to the family Herpesviridae, subfamily Alphaherpesvirinae, and genus Varicellovirus. Swine serve as both the natural and reservoir hosts of PRV; however, the virus has a broad host range and can infect various non-porcine mammals, including cattle, sheep, dogs, cats, rodents, and numerous wild species (2). In these non-natural hosts, PRV always leads to severe acute fatal neuropathy (3). Recent evidence suggests that, in rare cases, PRV is capable of cross-species transmission to humans, resulting in severe outcomes such as viral encephalitis and intraocular inflammation (4–7). In natural hosts, infections typically manifest with varying symptoms that correlate with the age stages of the pig herd. The primary symptoms observed include neurological disorders and high mortality rates in piglets, respiratory distress and weight loss in fattening pigs, and vertical transmission in sows, which can lead to abortion, stillbirth, and fetal mummification (8). Breeding boars may also suffer from infertility (9, 10).

Sow fertility is a critical determinant of economic efficiency in swine production, with the number of live piglets per litter serving as a key index of annual productivity. Consequently, the occurrence of reproductive disorders in sows has a direct and detrimental impact on the profitability of pig farms (11–13). Although live-attenuated vaccines against porcine PRV have been widely deployed and have significantly reduced disease incidence, the emergence of novel variant strains and the virus’s capacity to establish latent infections complicate sustained eradication efforts. In the event of an outbreak, the economic burden on the swine industry can be substantial. The potential public health threat posed by PRV also should not be overlooked. While human infections with PRV have thus far been limited to isolated cases of encephalitis and endophthalmitis, it is noteworthy that the herpes simplex virus (HSV) and varicella-zoster virus (VZV)—closely related members of the same Alphaherpesvirinae subfamily—are known to cross the placental barrier (PB) and cause congenital infections (14–18). Given the phylogenetic relatedness and shared virological characteristics among these viruses, the possibility of vertical transmission of PRV, although currently unconfirmed in humans, warrants heightened surveillance and further investigation. Existing mechanistic studies on PRV have predominantly focused on its neuroinvasive capacity, immune evasion strategies, and the molecular determinants of cross-species transmission (19, 20). While PRV is well-documented to cause fetal infection and intrauterine death, there remains a lack of research on the precise mechanisms of vertical transmission across the PB (21).

The placenta is a temporary yet essential organ that forms the interface between the mother and fetus during mammalian gestation. It serves as a central regulator of maternal physiological homeostasis, orchestrating resource allocation and supporting fetal development throughout pregnancy (22). Structurally, the placenta comprises a maternal component, a fetal component, and the PB—a specialized interface that facilitates material exchange between maternal and fetal circulations. This barrier consists of multiple cellular layers, including the basement membrane, trophoblasts, and fetal endothelial cells, which function collectively to mediate selective permeability, thereby playing a pivotal role in nutrient exchange, hormonal regulation, and immune defense (23). The placenta mediates the active transport of essential substrates such as glucose, amino acids, and fatty acids through specialized transporter proteins, ensuring an adequate supply of energy and biosynthetic precursors required for fetal growth and organogenesis (24, 25). Concurrently, it facilitates the removal of fetal metabolic waste, including carbon dioxide, into the maternal circulation. Beyond its metabolic functions, the placenta acts as a dynamic endocrine organ, synthesizing and releasing critical hormones such as progesterone, estrogens, and placental growth factor, which are indispensable for pregnancy maintenance, maternal physiological adaptation, and fetal development (26). The placenta serves as a selective immunological barrier, safeguarding the fetus from maternal immune rejection and pathogenic threats. It achieves this by secreting immunomodulatory molecules, attenuating maternal immune responses, and facilitating the transplacental transfer of maternal antibodies, thereby conferring passive immunity to the fetus. Under normal physiological conditions, the PB effectively restricts the passage of most toxins, infectious pathogens, and some pharmaceutical agents (27). However, a subset of pathogens can breach this barrier, leading to vertical (mother-to-child) transmission and potentially adverse pregnancy outcomes (28).

PRV can enter the bloodstream via leukocyte-mediated uptake, enabling systemic dissemination to multiple organs, including placental tissues, thereby resulting in stillbirth or abortion (29). A limited number of studies have indicated that the PRV may disseminate from primary replication sites to target organs, such as the gravid uterus, via cell-associated viremia within peripheral blood mononuclear cells (PBMCs). Subsequent secondary replication in the endothelial cells of the uterine vasculature has been associated with vasculitis and multifocal thrombosis, events believed to constitute the initial step in transplacental viral transmission and a plausible mechanism underlying PRV-induced miscarriages and fetal mummification (30). It has been proposed that PRV can infect placental cells through direct cell-to-cell transmission, entirely bypassing the extracellular phase (31). Recent evidence further demonstrates that, even in the presence of virus-neutralizing antibodies, PRV is capable of infecting endothelial cells of the placental vasculature through a CD11b/CD18-mediated adhesion and membrane fusion mechanism, implicating integrin-associated entry pathways in vertical transmission (32). However, the precise route by which PRV crosses the PB remains incompletely understood. It is yet to be determined whether PRV gains access to fetal compartments via active invasion of trophoblasts, cytoplasmic transport, or indirectly by inducing structural or functional disruption of placental integrity. Additionally, studies characterizing the spatiotemporal dynamics of PRV distribution across placental and fetal compartments are scarce, warranting further investigation.

To elucidate the pathophysiological basis of PRV-induced placental dysfunction and to gain a deeper understanding of the vertical transmission of PRV through the placenta, this study established a mouse model of placental infection in fetuses. First, we confirmed the damage and invasiveness of PRV to the placental barrier through hematoxylin-eosin (HE) staining and immunohistochemistry (IHC) analysis. Subsequently, we investigated the disruption of TJPs in the placental barrier caused by PRV using immunofluorescence (IF) staining and western blotting (WB) with specific marker proteins. Finally, we assessed the functional impairments caused by PRV infection at the gene level through transcriptomic analysis. This study elucidated the mechanisms underlying PRV-induced placental barrier disruption in mice from various aspects, including apoptosis, inflammatory processes, and hormone secretion. Through empirical research, we supported the vertical transmission of PRV through the placenta in mice and provided additional foundational references for understanding the mechanisms of maternal–fetal transmission of viral pathogens.

## MATERIALS AND METHODS

### Viruses

PRV XJ virus was isolated, identified, and stored in our laboratory. All virus stocks were propagated and titrated in BHK-21 cells using the 50% tissue culture infectious dose (TCID₅₀) assay. The titer of PRV-XJ was 10⁸ TCID₅₀/mL.

### Determination of LD_50_ in the intramuscular injection infection mouse model

The PRV XJ strain was diluted in a 10-fold gradient. Forty 6-week-old female Kunming mice were randomly divided into seven experimental groups (Groups A–G) and a control group (Group H), with five mice in each group. Each group was inoculated with different doses of the viral solution. The incidence and mortality rates of the mice were observed and recorded daily for 7 days following inoculation. The LD₅₀ was calculated to be 10 using the Reed-Muench method (Table 1).

**TABLE 1:** Result of median lethal dose of strain XJ by intramuscular injection

Dilution	Number	Death	Survival	Accumulated number of deaths	Accumulated number of survival	Total	Fatality rate (%)
10^−1^	5	5	0	20	0	20	100.0%
10^−2^	5	5	0	15	0	15	100.0%
10^−3^	5	5	0	10	0	10	100.0%
10^−4^	5	4	1	5	1	6	80.0%
10^−5^	5	1	4	1	5	6	20.0%
10^−6^	5	0	5	0	10	10	0.0%
10^−7^	5	0	5	0	15	15	0.0%

### Construction of the mouse infection model

Six-week-old Kunming mice were obtained from Beijing Huafukang Biotechnology Co., Ltd. and were housed in a room, which was maintained at a constant temperature of 25°C under a 12:12-h light–dark cycle. The experiment commenced 7 days after the mice acclimatized to their environment. During the course of the experiment, the subjects were provided with *ad libitum* access to food and water.

Following acclimatization, mice were randomly assigned to two groups (*n* = 20 per group): the control group and the PRV challenge group. Female and male mice were mated overnight at a 2:1 ratio, with the presence of a vaginal plug the following morning, indicating embryonic day 0.5 (E0.5). Upon observation of the vaginal plug, females were individually housed and maintained on the respective diet for the duration of gestation. Based on the previous LD_50_ test results, the pregnant females in the PRV group received an intramuscular injection of 0.2 mL of PRV suspension (5.5 × 10³ TCID₅₀/mL) on E12.5, while control animals received an equivalent volume (0.2 mL) of DMEM via the same route. After injection, PRV- and DMEM-treated mice were housed separately in temperature-controlled, well-ventilated rooms under a 12-h light/dark cycle.

After the injection of PRV, changes in body weight, clinical symptoms, and abortion rates of the mice in each group were observed and recorded daily. On E17.5, each group of mice was fasted for 12 h, and blood was collected via intra-orbital bleeding under pentobarbital sodium anesthesia and centrifuged. Serum samples were then separated and stored at −80°C until analysis.

The placentas were collected from the mice after humane euthanasia using an overdose of pentobarbitalum natricum. Samples intended for omics sequencing and validation experiments were stored at −80°C, while samples for histopathological section preparation were fixed in 4% paraformaldehyde.

### Gross and microscopic lesion analysis

Following complete tissue fixation, samples were processed through a graded ethanol series for dehydration, followed by paraffin embedding. The tissues were sectioned into 5-μm-thick paraffin slices using a standard microtome. The paraffin sections underwent standard processing steps, including dehydration, clearing, wax infiltration, embedding, and serial sectioning. The prepared sections were stained with H&E, followed by scanning using the Pannoramic SCAN II digital slide scanner (3DHISTECH, Budapest, Hungary). Digital images were analyzed using Image-Pro Plus 6.0 software (Media Cybernetics, Rockville, MD, USA). For image analysis, representative regions of interest (ROIs) were first identified based on histopathological features. Higher-magnification images were subsequently acquired from the same ROIs to allow detailed visualization and quantitative analysis.

### Measurement of viral burden

Total DNA was extracted from placental tissues and serum samples using the FastPure Viral DNA/RNA Mini Kit (Vazyme, China), in accordance with the manufacturer’s protocol. PCR was conducted to detect PRV DNA utilizing gE gene-specific primers: the forward primer 5′-CTTCCACTCGCAGCTCTTCT-3′ and the reverse primer 5′-TAGATGCAGGGCTCGTACAC-3′. The PCR thermocycling conditions were as follows: an initial denaturation at 95°C for 5 min; followed by 35 amplification cycles consisting of denaturation at 95°C for 1 min, annealing at 60°C for 1 min, and extension at 72°C for 90 s; concluding with a final extension at 72°C for 10 min. Viral loads were quantified by calculating the logarithmic value of the viral DNA copy number per 0.1 g of tissue, expressed as log₁₀ copies/0.1 g.

### IHC analysis

Immunohistochemical staining was conducted according to the aforementioned method (33), utilizing a rabbit monoclonal primary antibody targeting the PRV nucleocapsid protein (1:500 dilution; ABTC) and a biotin-conjugated affinity-purified goat anti-rabbit IgG secondary antibody (Proteintech, USA).

### IF analysis

IF was conducted on paraffin-embedded placental sections following heat-induced epitope repair. The sections were permeabilized using 0.1% Triton X-100 in PBS, followed by a blocking step with PBS containing 1% bovine serum albumin (BSA) and 0.3% Triton X-100. Subsequently, the sections were incubated overnight at 4°C with primary antibodies (ABclonal, China; all at a dilution of 1:100) against zonula occludens-1 (ZO-1) (A0659), occludin (A2601), placental growth factor (PLGF) (A2400), and proliferating cell nuclear antigen (PCNA) (A0264). Afterward, the sections were incubated with Alexa Fluor 488-conjugated donkey anti-rabbit IgG(H+L) (Thermo Fisher A21206), diluted at a ratio of 1:400, as the secondary antibody. Nuclei were counterstained with DAPI (1 µg/mL) for 10 min, and images were acquired using fluorescence microscopy.

### Western blot analysis

WB experiments were conducted in accordance with previous studies (1), utilizing primary antibodies specific to ZO-1 and occludin, PLGF, PCNA, and β-actin as an internal loading control, along with HRP goat anti-rabbit IgG(H+L) secondary antibodies.

### Terminal deoxynucleotidyl transferase dUTP nick end labeling (TUNEL) assay

Apoptotic cells in placental tissues were detected using the TUNEL FITC Apoptosis Detection Kit (Vazyme, China), in accordance with the manufacturer’s instructions. Following staining, TUNEL-positive cells were observed under a fluorescence microscope, and the apoptotic index was calculated as the percentage of apoptotic cells relative to the total number of cells in randomly selected microscopic fields.

### Detecting cytokines in the placenta and serum

Placental tissues were harvested and immediately placed in pre-chilled phosphate-buffered saline (PBS) at 4°C, followed by mechanical homogenization using a cold glass homogenizer. The homogenates were centrifuged at 3,000 rpm for 15 min at 4°C, and the resulting supernatants were collected into sterile Eppendorf tubes for the following analysis. Whole-blood samples were collected in serum-separation tubes without anticoagulants and allowed to clot at 25°C for 30 min. The samples were then centrifuged at 2,000 × *g* for 10 min at 4°C, and the resulting sera were collected for cytokine analysis. The concentrations of interleukin-1β (IL-1β), IL-4, IL-6, tumor necrosis factor-α (TNF-α), transforming growth factor-β (TGF-β), and interferon-γ (IFN-γ) were measured using commercial enzyme-linked immunosorbent assay (ELISA) kits (Thermo Fisher Scientific, USA), following the manufacturer’s protocols. Absorbance at 450 nm was measured using a microplate reader (Bio-Rad, USA), and cytokine concentrations were calculated based on standard curves.

### Detecting hormone secretion in the serum

Progesterone (P4) concentrations in the mouse serum were measured using a commercial enzyme-linked immunosorbent assay (ELISA) kit, following the manufacturer’s instructions.

### Transcriptome sequencing of placenta samples

#### RNA extraction library construction and sequencing and RNA-seq data analyses

Total RNA was extracted from placental tissues using TRIzol (Thermo Fisher, Cat. 15596018) per the manufacturer’s instructions. RNA quantity and purity were assessed on a NanoDrop ND-2000 (OD_260/280_ 1.8–2.2; OD_260/230_ ≥ 2.0), and integrity was confirmed with an Agilent 5300 Bioanalyzer (RIN > 7.0). Library preparation and sequencing were performed by Shanghai Majorbio Bio-pharm. Using 1 µg of high-quality RNA, mRNA was enriched via poly(A) selection (Illumina Stranded mRNA Prep Kit), fragmented, and reverse-transcribed into double-stranded cDNA (SuperScript kit). After end repair, A-tailing, and size selection (~300 bp), libraries were PCR-amplified (15 cycles), quantified by Qubit 4, pooled equimolarly, and sequenced (2 × 150 bp) on an Illumina NovaSeq X Plus.

#### Quality-control analysis and read mapping

Raw sequencing data were quality assessed and filtered using fastp (v0.23.2; HaploX, China), removing low-quality reads and adapter sequences based on standard metrics (e.g., per-base quality, GC content, and duplication levels). Cleaned reads were aligned to the *Mus musculus* reference genome (mm10) using HISAT2 (v2.2.1; Johns Hopkins University, USA) with default parameters.

#### Differential expression and functional-enrichment analysis

Differentially expressed genes (DEGs) were identified by quantifying transcript abundance using TPM normalization and estimating expression levels with RNA-seq by expectation maximization (RSEM). DESeq2 or DEGseq was applied for differential analysis, depending on the data set structure. DEGs were defined as those with |log₂(fold change)| ≥ 1 and FDR < 0.05 (DESeq2) or FDR < 0.001 (DEGseq). Functional enrichment analysis of DEGs was performed using GO and KEGG databases, with statistical significance defined by Bonferroni-corrected *P* < 0.05. GO enrichment was conducted with GOATOOLS and KEGG pathway analysis, with the SciPy library in Python.

#### Alternative-splicing events

Selective splicing events occurring in different samples were identified using the rMATS program. Only isoforms similar to the reference sequence or containing novel splice sites were considered, and splicing differences were detected based on exon inclusion, exon exclusion, 5′, 3′, and intron retention events.

#### Validation of the RNA-seq results by RT-qPCR

Six mRNAs were randomly selected to verify the accuracy of the RNA-seq data. Their expression levels were measured by quantitative real-time PCR (RT-qPCR), which was performed on total RNA (Invitrogen) extracted from the placental tissues of infected and control mice using TRIzol reagent. Reverse transcription was carried out using the PrimeScript RT Reagent Kit (Takara), and SYBR Green Master Mix (Sangon Biotech) was used for amplification. Oligo 7 version 7.60 software was employed to design the primers, which were synthesized by Sangon Biotech (Table 2). Relative mRNA expression levels were determined using the 2^−ΔΔCt^ method, and the data were log-transformed to log_2_ fold changes for better clarity and ease of interpretation.

**TABLE 2:** The primer sequences for qPCR

Gene	Primer	Sequence	Product size (bp)	Tm (°C)
prl3a1	F	GTCAACCATGTTCCTGGATGTT	83	60
	R	GCACATGGGCATGGAATACAC		
Occludin	F	CACACCTCGTCGCTAGTGC	122	60
	R	AGATAAGCGAACCTGCCGA		
smad3	F	AACGGGCAGGGAGAAGT	149	60
	R	CCATCCAGTGACCTGGGGAT		
cxcl1	F	CAAACCGAAGTCATAGCCACA	114	60
	R	CCGTTACTTGGGGACACCTTT		
CD14	F	CAGAATCTACCGACCATGGAGC	112	60
	R	TGCAGGAACAACTTTCCTCGT		
plac1	F	CAAGGAGTTCCATCTTAGCTGCC	119	60
	R	AGGAGACAAGAAGGCGTCCA		

#### Statistical analysis

All experimental data were analyzed using GraphPad Prism software and were expressed as the means ± standard deviation. A significance level of *P* < 0.05 was considered statistically significant.

## RESULTS

### PRV induces significant weight loss and fetal damage in pregnant mice

**Fig 1:**
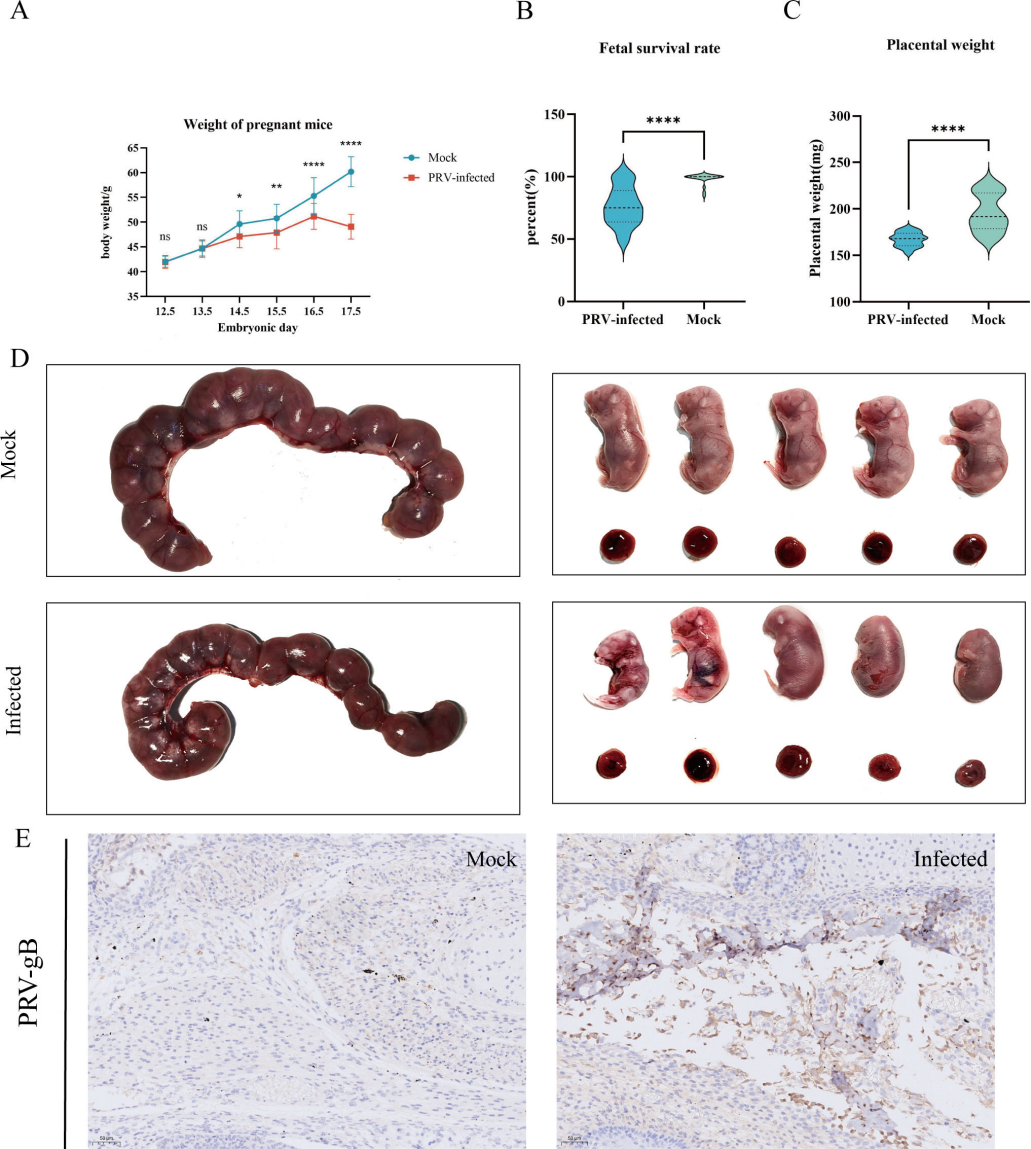
PRV induces significant weight loss and fetal damage in pregnant mice. (**A**) Daily weight changes in female mice after PRV infection during pregnancy. ****P < 0.0001; ***P < 0.001; **P < 0.01; *P < 0.05; ns, not significant. (**B**) Fetal survival on E17.5 after infection with PRV on E12.5. ****P < 0.0001. (**C**) Placental weight on E17.5. Data are representative of 5 independent experiments with one pregnant female dam per experiment, with four placentas randomly selected from each mouse for weighing. ****P < 0.0001. (**D**) Representative images of fetuses from pregnant mice in the PRV infection group and control group at E17.5. Fetuses in each group were obtained from the same representative dam. Fetuses in the PRV infection group showed growth restriction. (**E**) Representative image of the head tissues of a fetal mouse at E17.5 that were immunostained with anti-PRV-gB antibody. (Scale bar, 50 μm.)

Following the PRV challenge, pregnant female mice in the infected group exhibited a significant reduction in average daily weight gain compared to the control group, with a pronounced decline observed shortly before necropsy (Fig. 1A). Although no abortions occurred, a subset of infected dams succumbed, and both stillbirths and gross fetal malformations were documented. The embryo survival rates for each group are illustrated in Fig. 1B. Notably, PRV infection resulted in an approximately 10% decrease in mean placental weight relative to controls (Fig. 1C), suggesting compromised placental function. A macroscopic morphological examination of the fetus was performed. Several fetuses in the PRV group exhibited features consistent with mild intrauterine growth restriction (IUGR), including reduced body size, incomplete craniofacial formation, aberrant limb positioning, and delayed skin keratinization (Fig. 1D). To ascertain whether viral transmission to the fetus occurred, immunohistochemical staining for PRV antigen was performed on fetal brain tissue. Strong positive PRV signals were detected in the brain sections of infected fetuses, whereas no detectable antigen was present in control samples, indicating direct fetal infection via transplacental transmission (Fig. 1E).

### PRV induces abnormal placental development

**Fig 2:**
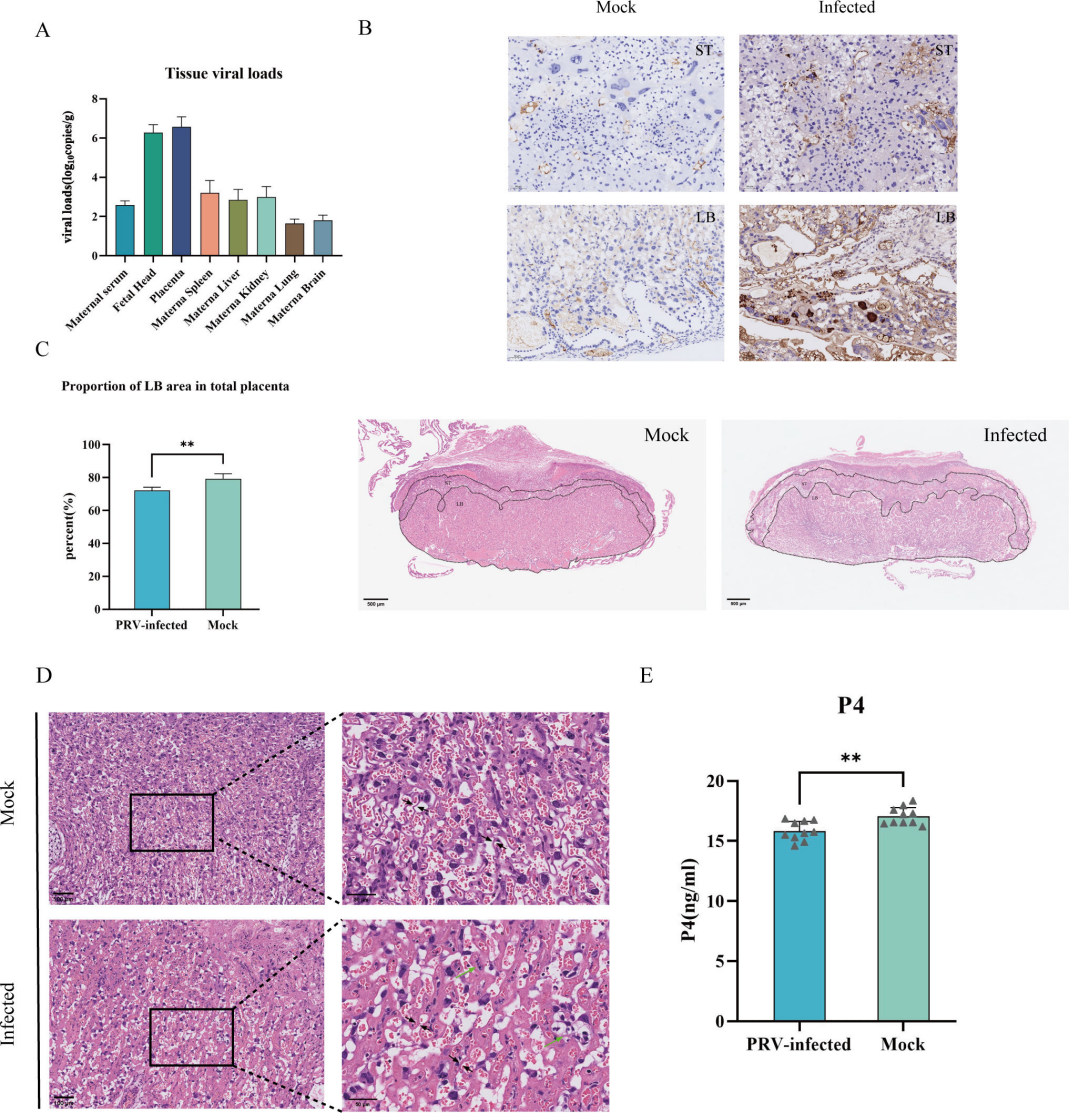
PRV induces abnormal placental development and dysregulation of progesterone secretion. (**A**) Viral burden was measured by q-PCR assay from maternal serum, fetal head, placenta, maternal spleen, maternal liver, maternal kidney, maternal lung, and maternal brain at E17.5. The data are from individual animals and are averages from 10 independent experiments. (**B**) Representative images of histological sections of PRV-infected placentas that were immunostained with an anti-PRV-gB antibody. ST, spongy trophoblast layer; LB, labyrinth layer. (Scale bar, 50 μm.) (**C**) PRV infection causes a reduction in the area of the labyrinth zone of the placenta in mice. The left panel shows the proportion of the labyrinth zone to the total placental area. The right panel shows representative histological images of the placenta stained with hematoxylin and eosin at E17.5. (Scale bar, 500 μm.) **P < 0.01. (**D**) Representative hematoxylin and eosin staining showed pathological features of placentas at E17.5. The right panel shows a higher-magnification view of the boxed area in the left panel. Green arrows indicate apoptotic trophoblasts. The black arrow indicates thickened vascular interstitium. (Scale bar, 100 μm, 50 μm.) (**E**) P4 levels in the serum of the indicated female mice were quantified by ELISA on E17.5 (*n* = 10). **P < 0.01.

To further investigate whether PRV causes fetal infection through vertical transmission, we employed fluorescent quantitative real-time polymerase chain reaction (RT-qPCR) to quantify PRV levels in various maternal tissues and organs, as well as in the fetal head. The results indicated that high viral loads were detected in the placenta and fetal brain tissues, with levels significantly exceeding those found in maternal serum and other maternal organs (Fig. 2A). Immunohistochemical staining demonstrated significant PRV-positive signals in the placentas of the infected group, primarily localized in the labyrinth zone adjacent to the fetal side, while the junction zone exhibited only minimal positive signals. In contrast, the placentas from the control group displayed negative reactions to PRV antigens (Fig. 2B).

Analysis of placental tissue histopathological sections revealed altered proportions of the junctional zone and labyrinth zone in the placentas of infected mice (Fig. 2C). Notably, there was a reduction in the area of the labyrinth zone, which serves as the primary site for material exchange and nutrient supply in the placenta. The abnormal placental tissue structure was characterized by disorganization of junctional cells, with areas exhibiting necrosis. Additionally, a small number of apoptotic cells contributed to the formation of calcification foci. The labyrinth zone displayed increased villous branching, indicating severe vascular damage characterized by the destruction of fetal capillaries and placental microvessels. Irregularities and narrowing of maternal blood sinuses, thickening of the vascular intermembrane, and abnormal morphology of trophoblast cells were also observed (Fig. 2D).

Progesterone is essential for maintaining uterine quiescence. Disturbances in its biosynthesis or signaling pathways are implicated in the pathogenesis of preeclampsia and spontaneous abortion (34). In the present study, we observed slightly but significantly decreased progesterone concentrations in maternal serum from PRV-infected dams compared to controls (Fig. 2E). Insufficient progesterone levels may affect the proliferation of trophoblast cells, leading to vascular damage and ultimately increasing the risk of fetal miscarriage (35). Insufficient progesterone levels may impair trophoblast proliferation, lead to abnormal placental morphogenesis, reduce vascular remodeling, and ultimately increase the risk of fetal loss.

### PRV induces impaired placental barrier function

**Fig 3:**
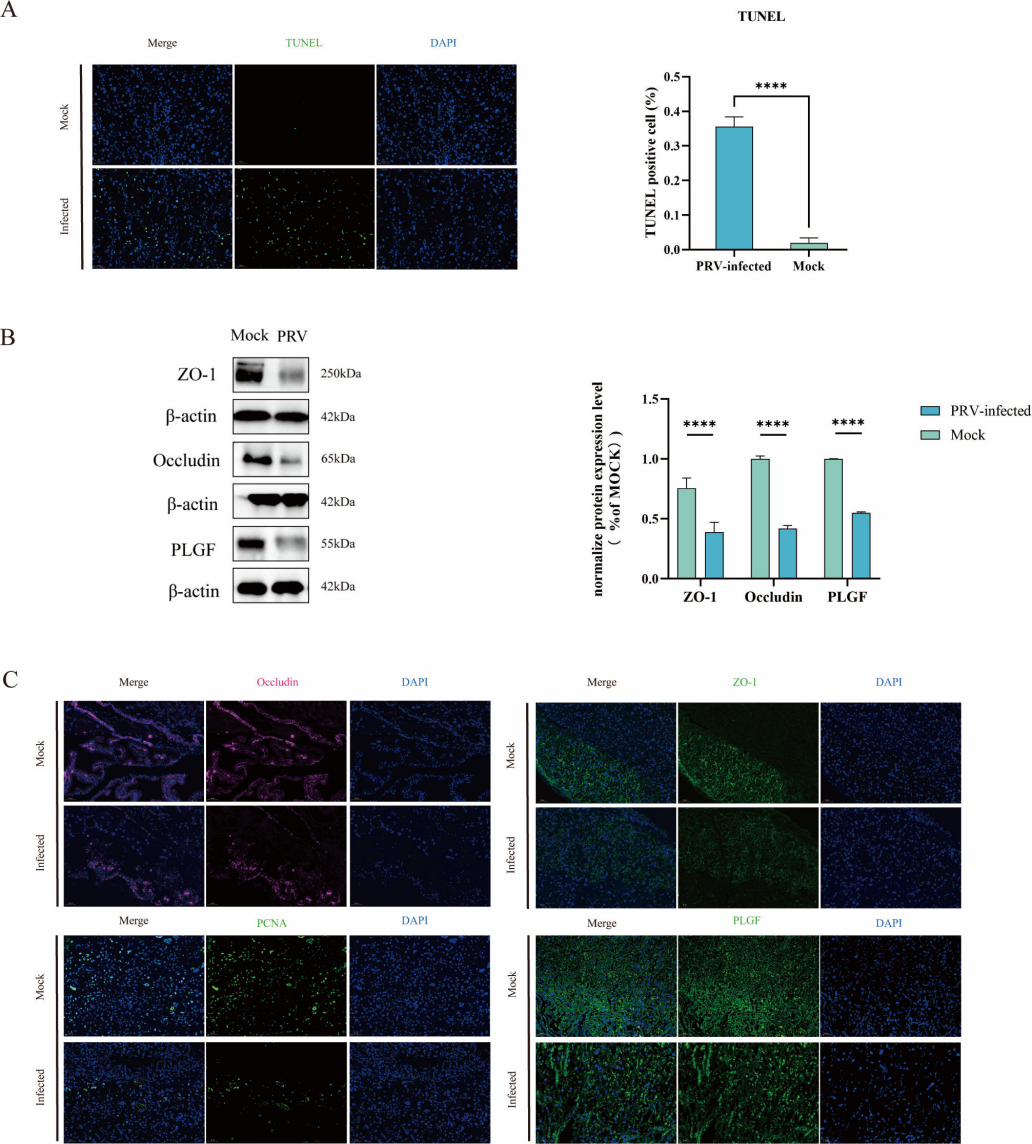
PRV induces impaired placental barrier function. (**A**) Detection of apoptosis in placental cells by performing TUNEL assays. The right panel shows the proportion of TUNEL-positive cells in the statistics. (Scale bar, 50 μm.) ****P < 0.0001. (**B**) WB depicted that the amount of ZO-1, occludin, and PLGF in the placenta decreased after PRV infection. The levels of ZO-1, occludin, and PLGF were normalized by β-actin, and the comparison with the mock group was quantified in the right panel. ****P < 0.0001. (**C**) IF staining for ZO-1, occludin, PLGF, and PCNA expression in mouse placental sections (scale bar, 50 μm).

Trophoblast cells are one of the principal functional components of the placenta, essential for the successful establishment and maintenance of pregnancy. These cells are in direct contact with maternal blood and form the structural and functional foundation of the maternal–fetal interface. In addition to facilitating the bidirectional exchange of gases, nutrients, and waste products, trophoblasts actively participate in shaping the local immune environment and regulating endocrine signals critical for fetal development (36, 37). Given their central role, the apoptosis of trophoblast cells can severely compromise PB integrity and impair overall placental function. To investigate whether PRV infection induces apoptosis in placental cells, TUNEL assays were conducted on paraffin-embedded placental sections. The results revealed a significantly elevated number of TUNEL-positive cells in the infected group compared to the control group, with apoptotic signals predominantly localized to the labyrinth zone—the key site for maternal–fetal exchange (Fig. 3A). This may be one of the causes of impairment of PB function.

TJPs, including ZO-1 and occludin, are critical components of the PB that regulate paracellular permeability and maintain the structural and functional integrity of the maternal–fetal interface (38). Disruption or downregulation of TJPs can compromise barrier function, thereby increasing placental permeability and facilitating the translocation of pathogens, inflammatory mediators, and other harmful substances into the fetal compartment. PRV may exploit this mechanism to traverse the PB and invade fetal tissues. To evaluate this hypothesis, we first assessed the protein expression levels of ZO-1 and occludin using WB. Compared to the control group, PRV-infected placental tissues exhibited a marked reduction in the expression of both TJPs (Fig. 3B), suggesting compromised tight junction integrity. To further investigate whether PRV infection altered the spatial organization of these proteins, we performed IF staining to examine their localization within placental tissue. In the control group, ZO-1 and occludin displayed a continuous, well-defined distribution along the cell borders, whereas in the infected group, the fluorescence signals were notably weakened and appeared fragmented and discontinuous (Fig. 3C). These findings are consistent with the immunoblotting results and support the conclusion that PRV infection disrupts tight junction structure and function, thereby contributing to PB dysfunction.

Placental growth factor (PLGF) is a key angiogenic cytokine that promotes the development and remodeling of placental vasculature. Its dysregulation has been implicated in pregnancy-related disorders, such as preeclampsia (39). Proliferating cell nuclear antigen (PCNA), a nuclear protein involved in DNA replication and repair, serves as a marker of cellular proliferation. Both PLGF and PCNA contribute to the maintenance of PB integrity by supporting vascularization and trophoblast cell renewal, processes essential for sustaining fetal development and maternal–fetal exchange. To evaluate the impact of PRV infection on placental angiogenesis and cell proliferation, we performed indirect IF staining to examine the distribution and intensity of PCNA and PLGF signals in placental tissue sections. Additionally, PLGF expression levels were validated by WB analysis. The results demonstrated a marked reduction in both PCNA and PLGF fluorescence signals in the PRV-infected group compared to controls (Fig. 3C), indicating impaired proliferative capacity and angiogenic function within the placenta. Consistent with these observations, immunoblotting confirmed a significant downregulation of PLGF protein levels following PRV infection (Fig. 3B). These findings suggest that PRV-mediated disruption of proliferative and angiogenic signaling may contribute to placental dysfunction and adverse pregnancy outcomes.

### PRV causes a severe inflammatory response in the placenta

**Fig 4:**
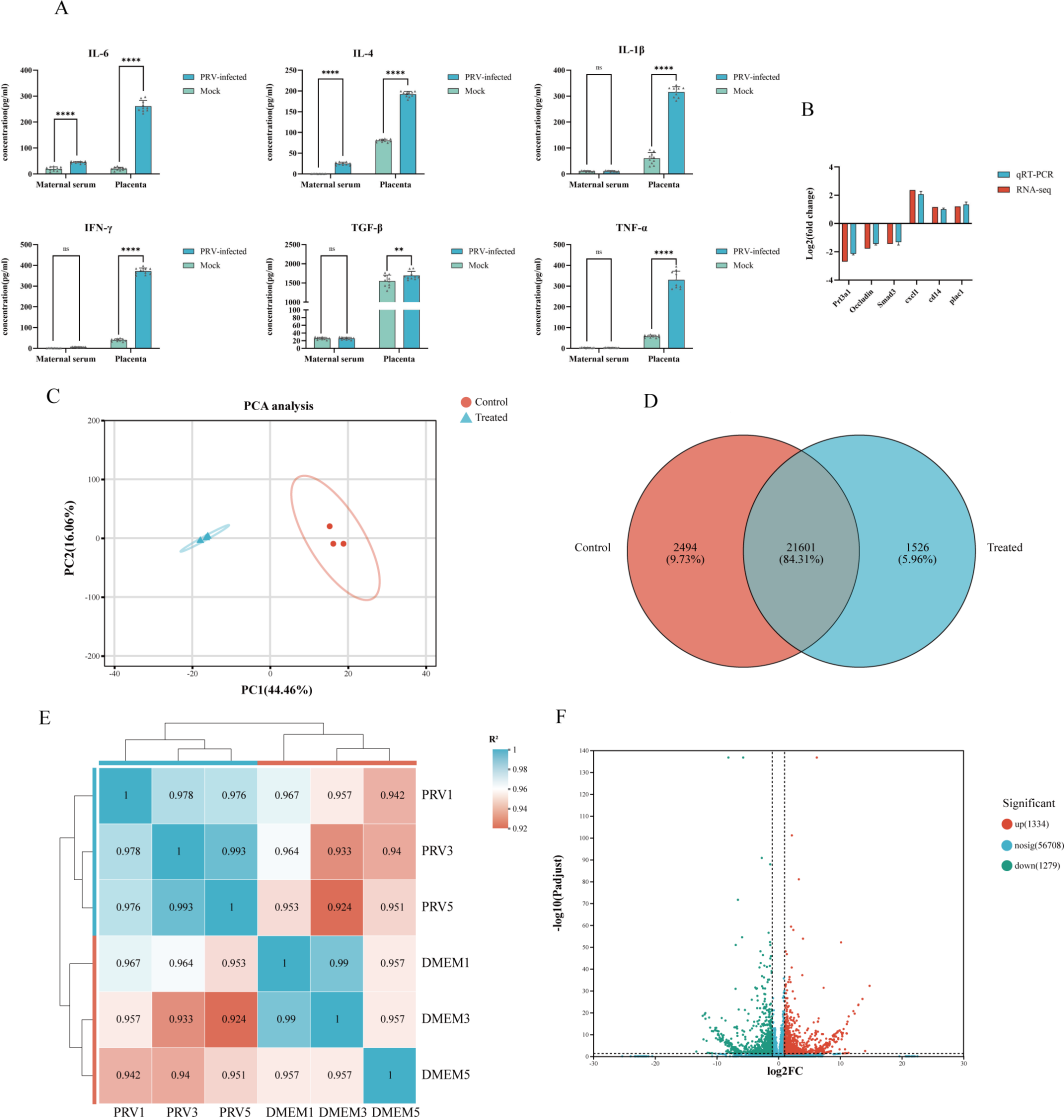
PRV causes a severe inflammatory response in the placenta. (**A**) The expression levels of IL-6, IL-4, IL-1β, IFN-γ, TGF-β, and TNF-α in the placenta were determined by ELISA analysis. ****P < 0.0001; **P < 0.01; ns, not significant. (**B**) Red and blue bars represented the whole transcription sequencing results and qRT-PCR validation results, respectively, whereas qRT-PCR validation results were displayed as mean log_2_ (fold change) ± SD with error bars from three independent experiments. (**C**) The principal components analysis of differential expression genes. The ellipses represent the confidence intervals for each group. (**D**) Venn diagram illustrating the number of detected mRNAs. (**E**) Heat map based on Pearson correlation analysis. (**F**) Volcano plots were used to identify significantly different mRNA transcripts under different biological conditions.

To evaluate the impact of PRV infection on placental inflammation, we analyzed cytokine expression profiles. The results revealed a significant upregulation of IL-6, IFN-γ, IL-1β, and TNF-α in placental tissues from the PRV-infected group, indicative of a robust local inflammatory response (Fig. 4A). These findings suggest that PRV infection elicits innate immune activation within the placenta, potentially disrupting the immune-tolerant microenvironment essential for fetal development.

To investigate changes in placental gene expression following PRV infection, RNA sequencing was conducted on placental tissues collected from three mice per group at embryonic day 17.5 (E17.5). A total of six libraries generated 96.38 Gb of data, with each sample yielding over 14.71 Gb of high-quality clean reads and Q30 base percentages exceeding 94.57% (Table 3). Six selected DEGs were further validated by RT-qPCR in placental tissues, and the observed expression patterns were consistent with the RNA-seq results (Fig. 4B). Principal component analysis (PCA) demonstrated clear separation between groups and tight clustering within groups, indicating both high intragroup consistency and significant intergroup differences (Fig. 4C). Venn diagram analysis illustrated the number of detected mRNAs and their shared and unique expression across groups, confirming the feasibility of further differential expression analysis (Fig. 4D). Pearson correlation coefficients indicated strong reproducibility among biological replicates within each treatment group, with values exceeding 0.957 and 0.976 (Fig. 4E). In total, 2,613 differentially expressed genes (DEGs) were identified, comprising 1,334 upregulated and 1,279 downregulated mRNAs (Fig. 4F).

**TABLE 3:** Statistical analysis of the mRNA-sequencing data

Sample	Raw reads	Raw bases	Clean reads	Clean bases	Error rate (%)	Q20 (%)	Q30 (%)	GC content (%)
PRV1	108,920,564	16,447,005,164	107,538,890	15,889,796,848	0.0128	98.25	94.65	55.5
PRV3	115,050,314	17,372,597,414	113,705,718	16,821,161,284	0.0128	98.27	94.72	54.89
PRV5	116,374,870	17,572,605,370	114,896,602	16,904,405,835	0.0128	98.22	94.57	55.18
DMEM1	100,150,118	15,122,667,818	99,068,130	14,713,221,589	0.0127	98.29	94.76	53.11
DMEM3	114,391,172	17,273,066,972	113,163,600	16,809,478,267	0.0126	98.34	94.91	52.84
DMEM5	104,822,342	15,828,173,642	103,575,998	15,237,917,670	0.0126	98.36	94.97	53.32

**Fig 5:**
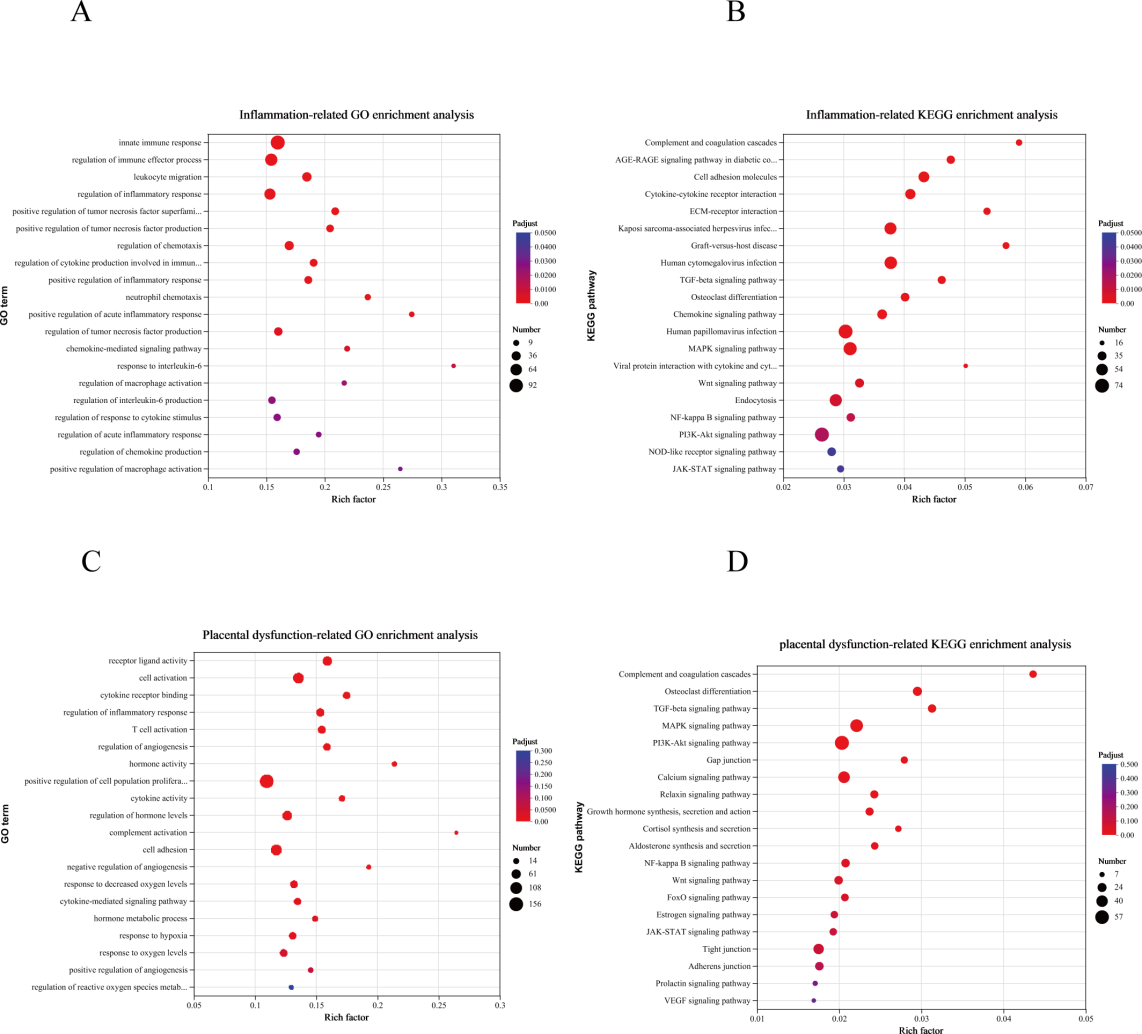
Potential placental dysfunction due to PRV. (**A**) GO-enrichment analysis of dif-mRNAs associated with inflammation. The top 20 GO pathways are shown. (**B**) KEGG enrichment analysis of dif-mRNAs associated with inflammation. The top 20 KEGG pathways are shown. The sizes and colors of the solid circles represent the number of enriched dif-mRNAs and the significance of the enrichment, respectively. (**C**) GO enrichment analysis of dif-mRNAs associated with potential placental dysfunction. The top 20 GO pathways are shown. (**D**) KEGG-enrichment analysis of dif-mRNAs associated with potential placental dysfunction. The top 20 KEGG pathways are shown. The sizes and colors of the solid circles represent the number of enriched dif-mRNAs and the significance of the enrichment, respectively.

Transcriptome sequencing results also indicated that inflammatory pathways were significantly activated in the PRV infection group. We identified 20 Gene Ontology (GO) and Kyoto Encyclopedia of Genes and Genomes (KEGG) terms that are believed to be involved in placental inflammatory responses (Fig. 5A and B). Gene Ontology (GO) enrichment analysis revealed significant activation of inflammation-related biological processes, including the regulation of macrophage activation (GO:0043030 and GO:0043032), chemokine production (GO:0050920, GO:0070098, and GO:0032642), and acute inflammatory responses (GO:0050727, GO:0050729, GO:0002675, and GO:0002673). Additional enriched terms included cytokine-mediated signaling pathways (GO:0002718 and GO:0060759), regulation of interleukin-6 biosynthesis (GO:0070741 and GO:0032675), neutrophil chemotaxis (GO:0030593), positive regulation of tumor necrosis factor production (GO:1903557, GO:0032760, and GO:0032680), leukocyte migration (GO:0050900), and activation of the innate immune response (GO:0045087). Collectively, these results indicate that PRV infection triggers a hyperinflammatory state in the placenta, characterized by IL-6- and TNF-α-centered signaling cascades, macrophage and neutrophil recruitment, and enhanced leukocyte trafficking via chemokine-mediated pathways. This inflammatory milieu may disrupt the immune-tolerant environment of the placenta, which is essential for maintaining pregnancy, and contribute to adverse fetal outcomes.

The Kyoto Encyclopedia of Genes and Genomes (KEGG) pathway enrichment analysis further elucidated a significant activation of inflammation-related signaling cascades within placental tissues following PRV infection. Notably, key immune regulatory pathways—including the NF-kappa B signaling pathway (map04064), cytokine–cytokine receptor interaction (map04060), chemokine signaling pathway (map04062), and JAK–STAT signaling pathway (map04630)—were significantly enriched. These pathways serve as central mediators of immune cell recruitment, pro-inflammatory cytokine production, and cellular stress responses, collectively indicating robust immune activation and potential infiltration of inflammatory cells within the placental microenvironment. In addition to classical immune signaling, the osteoclast differentiation pathway (map04380) was significantly enriched. Although osteoclasts are absent in placental tissues, key components of this pathway—such as the RANKL/NFATc1 axis, tumor necrosis factor (TNF), and reactive oxygen species (ROS)—are well-documented mediators of macrophage activation and tissue immunomodulation. The activation of this pathway indicates a heightened state of monocyte–macrophage activation within the placenta, contributing to localized inflammation and elevated oxidative stress, both of which may compromise placental structure and function. Furthermore, significant enrichment of the cell adhesion molecules (CAMs) pathway (map04514) suggests potential alterations in the structural integrity of the maternal–fetal interface. In placental tissues, adhesion molecules such as intercellular adhesion molecule-1 (ICAM-1) and vascular cell adhesion molecule-1 (VCAM-1) are critical for maintaining the stability of the chorionic villous membrane and regulating leukocyte adhesion and transmigration. Aberrant expression of these molecules may weaken the PB, increase vascular permeability, and facilitate the abnormal migration of maternal immune cells into fetal compartments, thereby raising the risk of inflammatory injury to fetal tissues.

### Potential placental dysfunction due to PRV

We selected 20 GO terms and 20 KEGG terms that may be related to placental dysfunction for display (Fig. 5C and D). GO enrichment analysis showed that differentially expressed genes in dysfunctional placentas were significantly enriched in immune-related processes, angiogenesis-associated pathways, oxidative stress-related responses, and hormone-related signaling. Enriched immune-associated terms included cell activation (GO:0001775), T-cell activation (GO:0042110), cytokine receptor binding (GO:0005126), cytokine activity (GO:0005125), and modulation of inflammatory response (GO:0050727). Angiogenesis and cell adhesion-related terms were also significantly enriched. In addition, enrichment was observed for hypoxia- and oxidative stress-related terms, including response to hypoxia (GO:0001666), response to decreased oxygen levels (GO:0070482), and regulation of reactive oxygen species metabolic process (GO:2000377). Hormone-related terms, such as hormone activity (GO:0005179), hormone metabolic process (GO:0042445), and regulation of hormone levels (GO:0010817), were also significantly enriched.

KEGG pathway enrichment analysis showed that differentially expressed genes were significantly enriched in multiple signaling pathways associated with placental biological processes. These included PI3K–Akt (map04151), MAPK (map04010), TGF-β (map04350), NF-κB (map04064), JAK–STAT (map04630), and FoxO (map04068) signaling pathways. In addition, pathways related to hormone synthesis and secretion were significantly enriched, including estrogen (map04915), prolactin (map04917), cortisol (map04927), growth hormone (map04935), and relaxin (map04926) signaling pathways.

Enrichment was also observed for the complement and coagulation cascades pathway (map04610). Furthermore, several pathways associated with cellular junctions and connectivity, including tight junction, adherens junction, and gap junction pathways, were significantly enriched.

GO and KEGG analyses collectively revealed that PRV-induced placental dysfunction encompasses various aspects, including immune signaling, inflammatory abnormalities, vascular development and injury, oxidative stress adaptation, and hormonal signaling disorders. These are all potential factors that compromise the integrity of the placenta and lead to adverse pregnancy outcomes.

## DISCUSSION

Many previous studies have shown that PRV can cause abortion and delivery of mummified fetuses in pregnant sows (40, 41), but no study has systematically elucidated the mechanism of vertical transmission of the virus across the PB. Considering the profound consequences of PRV infection, there is an urgent need for reliable small animal models to study PRV transmission during gestation. In this study, we established a mouse model of PRV infection during pregnancy and clearly demonstrated that pseudorabies virus (PRV) can directly infect the placenta and cross the PB to infect the fetus, which can lead to IUGR, fetal malformations, and serious consequences such as stillbirth. These models would allow for a systematic investigation of the mechanisms underlying vertical transmission, thereby identifying potential therapeutic targets to prevent or reduce intrauterine infections and ultimately block maternal–fetal transmission.

Vertical transmission includes various routes such as intrauterine infection, transmission through the birth canal during delivery, and postnatal transmission via breast milk. Both HSV and cytomegalovirus (CMV), which also belong to the herpes virus family as PRV, have been confirmed to be transmissible to newborns. However, reports of placental infection by HSV are rare, with most cases arising from exposure to HSV during delivery via the genital tract (14, 42). This may be attributed to the low frequency of viremia associated with primary genital HSV infections, while recurrent HSV infections almost never report viremia. In contrast, CMV is more likely to induce viremia, making the vertical transmission pattern through the placenta more common (43). In this study, the presence of viral DNA was detected in maternal blood, and the viral load in the placenta was significantly higher than that in the blood, indicating that viremia is an important aspect of PRV vertical transmission and that the virus preferentially replicates within the placenta. This observation is consistent with previous findings regarding Zika virus infection in pregnant mice (44). HE and IHC results indicate that viral infection causes significant pathological damage to the placental labyrinth, with PRV-positive signals detected in the placenta. Positive signals of PRV are predominantly localized in the decidual zone of the placenta and the labyrinth zone adjacent to the fetus, with only minimal positive signals observed in the junction zone. In contrast, the Zika virus is detected at lower levels within both the mononuclear trophoblast and the syncytial trophoblast (44). The distinct placental tropism of PRV and ZIKA may arise from their differing mechanisms of immune evasion. Furthermore, host resistance to viral transplacental migration may be influenced by the regulation of virus-specific receptors, which vary with cell differentiation, thereby contributing to the complexity and diversity of placental viral infections (45).

TJPs in the placenta are essential structural components of the maternal–fetal barrier. These proteins not only maintain the integrity of the intercellular barrier and regulate signal transduction but also serve as key connectors between the membrane and the cytoskeleton (23, 46), thereby influencing cellular polarity (47). Previous studies have shown that Zika virus infection in mice results in the downregulation of TJPs in the placenta, facilitating vertical transmission of the virus (48). A similar abnormality of TJPs has been observed in various disease models, including inflammatory conditions, high-fat diets, and gestational diabetic pregnancies, all of which indicate disruption of the maternal–fetal barrier (49, 50). To further investigate this phenomenon, we assessed the expression of ZO-1 and occludin, key components of tight junctions, using immunofluorescence (IF) and Western blotting (WB). The results revealed a significant reduction in the expression of ZO-1 and occludin in the placentas of infected mice, accompanied by structural disruption of ZO-1. Trophoblast giant cells express various vascular growth factors, potentially establishing a complex regulatory system that underpins placental vascular growth and maintenance. Inadequate vascular formation or maintenance within the placenta can result in compromised placental growth, dysfunction, hypoxia, fetal malnutrition, and may even lead to intrauterine fetal death (36, 51–53). To comprehensively evaluate placental growth and vascularization, we also examined the expression of proliferating cell nuclear antigen (PCNA) and placental growth factor (PLGF). IF analysis showed that PRV infection significantly decreased the expression of both markers, suggesting impaired cellular proliferation and inhibited angiogenesis, thus affecting developmental processes. The results of the TUNEL assay indicate a significant increase in apoptotic signals in the PRV-infected group, with positive signals primarily concentrated in the labyrinth region, which aligns with previous studies (54), providing further insights into the observed damage.

In mice, progesterone is primarily produced by the corpus luteum throughout gestation (55, 56), with its synthesis regulated by prolactin secreted by the placenta (57). In this study, we observed a slight yet statistically significant decrease in maternal serum progesterone levels in the PRV-infected group compared to the control group. The magnitude of this change is small, and its actual biological implications within this model warrant cautious interpretation. Therefore, we propose that altered progesterone levels may not be a decisive factor in PRV-induced adverse pregnancy outcomes but rather represent an endocrine disturbance accompanying infection. Consistent with the changes in serum levels, transcriptomic analysis revealed downregulation of certain genes associated with progesterone biosynthesis (e.g., HSD3B1 and members of the Prl family), suggesting that PRV infection may exert some influence on the endocrine synthetic function of the placenta. However, whether these molecular-level alterations are sufficient to cause functional progesterone deficiency warrants further investigation. Previous studies indicate that progesterone inhibits the expression of proinflammatory cytokines (such as IL-1β and IL-6) in mouse trophoblast cells (58). Therefore, even limited changes in progesterone levels may exert regulatory effects on the placental microenvironment within an inflammatory context. Moreover, we identified significant enrichment in signaling pathways associated with hormone synthesis and secretion, specifically estrogen, prolactin, cortisol, growth hormone, and relaxin pathways. These findings suggest that PRV infection may be associated with a global disruption of the placental endocrine regulatory network rather than a marked alteration in a single hormone level. Overall, in this mouse model, progesterone-related changes are more likely to represent one component of the multifactorial pathological process triggered by PRV infection rather than its primary driver.

To better understand the mechanisms by which PRV induces placental inflammatory responses, we measured cytokine levels in placental tissue. The results revealed that PRV infection significantly upregulates the expression of several cytokines, including IL-6, IL-1β, IL-4, TGF-β, TNF-α, and IFN-γ, indicating that the placenta is in a state of heightened inflammation and immune dysregulation. Previous studies have demonstrated that inflammation during pregnancy can impair placental development, leading to placental dysfunction (33, 59). For instance, TNF-mediated monocyte chemotactic protein released from the extrachorionic trophoblast layer may further amplify pro-inflammatory cascades, while TNF can inhibit trophoblast invasion *in vivo* (60, 61). Additionally, studies have shown that IFN-γ expression is significantly elevated in placental vascular endothelial cells during preeclampsia. This increase is associated with placental vascular stenosis, reduced uteroplacental blood flow, and placental hypoxia, suggesting that IFN-γ-mediated immune-inflammatory responses may interfere with normal angiogenesis and vascular remodeling, thereby exacerbating placental perfusion insufficiency (62).

To investigate the genetic abnormalities induced by PRV infection in the placenta, we conducted transcriptomic sequencing. The results of Gene Ontology (GO) and Kyoto Encyclopedia of Genes and Genomes (KEGG) pathway analyses indicated that differentially expressed genes were significantly enriched in biological processes such as the regulation of the acute inflammatory response, leukocyte chemotaxis, and macrophage activation. Furthermore, these genes were involved in key inflammatory signaling pathways, including NOD-like receptors, NF-κB, JAK–STAT, and chemokines. The recruitment of inflammatory cells, primarily macrophages and neutrophils, adversely impacts fetal development and elevates the risk of maternal miscarriage. Additionally, GO and KEGG analyses highlighted terms related to angiogenesis, including the regulation of angiogenesis, cell adhesion, and ECM receptor interaction pathways, alongside the downregulation of genes involved in vascular remodeling, such as Col5a1, Col8a1, Lamc2, Flrt1, and Smad2/3. Certain integrin receptors on the cell surface bind to ECM components, including fibronectin, laminin, and collagen, thereby promoting the adhesion and migration of vascular endothelial cells, which supports the formation of placental vessels. Based on these observations, we hypothesize that the placental blood supply network may be affected following PRV infection. However, transcriptomic analyses have inherent limitations, and these potential alterations require further functional validation.

Although our study establishes a murine model of maternal–fetal PRV transmission and elucidates mechanisms by which PRV disrupts placental barrier integrity, important anatomical and physiological differences between murine and porcine placentas should be considered when extrapolating these findings to pigs, the natural host of PRV. The porcine placenta is epitheliochorial, with multiple cellular layers strictly separating maternal and fetal blood supplies, thereby limiting trophoblast invasiveness and reducing permeability at the maternal–fetal interface (63). In contrast, mice possess a hemochorial placenta, in which fetal trophoblasts are directly exposed to maternal blood, providing a more permissive interface for viral access and immune interactions. These structural differences are likely to influence viral entry routes, transplacental transmission efficiency, and immune responses at the maternal–fetal interface. For instance, the hemochorial placenta in mice may facilitate viral access to trophoblasts via the maternal circulation, potentially exacerbating barrier disruption and fetal exposure. Conversely, the limited invasiveness and low permeability of the porcine epitheliochorial placenta may require viruses to employ more extensive or distinct strategies to breach the maternal–fetal barrier; for example, porcine reproductive and respiratory syndrome virus infection of the porcine placenta during fetal transmission has been associated with placental hypoxic stress, induced upregulation of virus receptor expression, and aberrant barrier remodeling (64–66). Accordingly, while the murine model captures key features of PRV-induced placental injury—including barrier dysfunction, inflammatory activation, and vascular damage—these findings should be interpreted with caution in the context of PRV pathogenesis in pigs. Future validation in porcine trophoblast-based systems or *in vivo* models will be essential.

Nevertheless, mice remain a crucial model organism for studying PRV. The pregnancy mouse model developed in this study provides a comprehensive understanding of the mechanisms by which PRV crosses the PB and undergoes vertical transmission *in vivo*. This process involves apoptosis, altered barrier permeability, endocrine dysfunction, oxidative stress, and pronounced inflammatory responses. Vertical transmission of herpesviruses has been well-documented in previous studies. While pseudorabies, as a potential zoonotic disease, currently represents a significant threat primarily within the livestock industry, it also poses potential public health risks that cannot be underestimated. Our findings may offer new insights into the mechanisms by which the PB prevents herpesvirus infection.

## Data Availability

The data that support the findings of this study are openly available in Zenodo at https://doi.org/10.5281/zenodo.17059759, reference number 17059759. The transcriptomic (RNA-seq) data supporting the findings of this study have been deposited in the NCBI database under accession number PRJNA1283603.
